# Supplementary material for: An Intricate Network Involving the Argonaute ALG-1 Modulates Organismal Resistance to Oxidative Stress
Source: Nat Commun. 2024 Apr 9;15:3070. doi: 10.1038/s41467-024-47306-4 (PMC11003958; doi:10.1038/s41467-024-47306-4)
Supplement: Supplementary file 1 — Supplementary Information [file 41467_2024_47306_MOESM1_ESM.pdf]

SUPPLEMENTARY INFORMATION

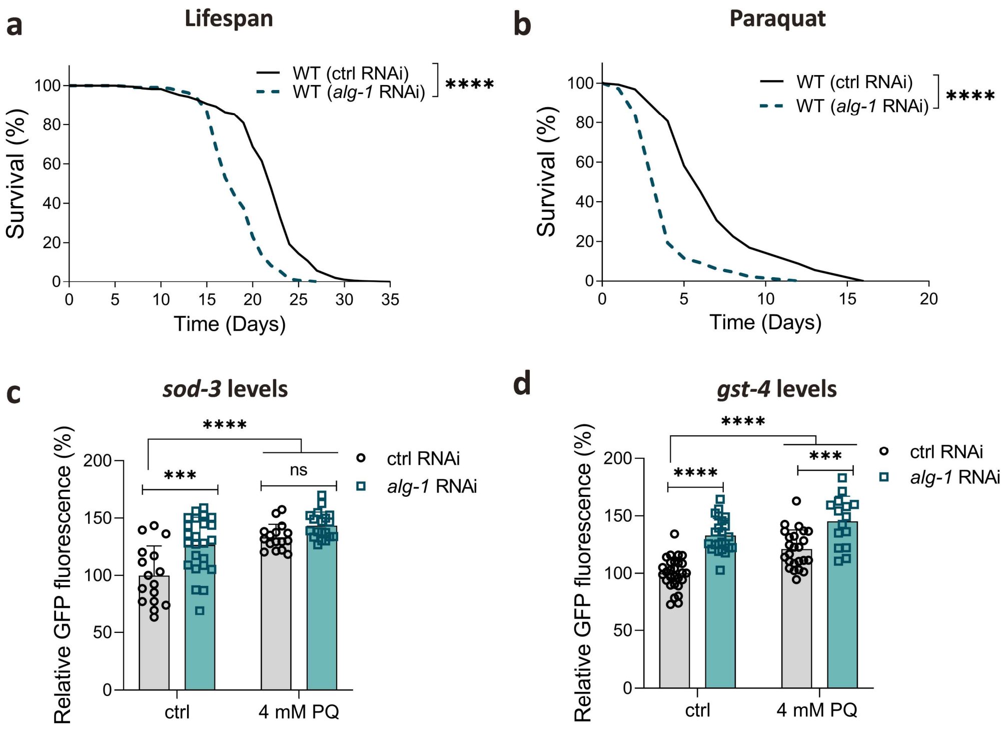

Supplementary Figure 1. *alg-1* knockdown in adults reduces *C. elegans* lifespan and oxidative stress resistance. (a) Lifespan of WT worms exposed to *alg-1* or control RNAi (empty vector) since day 0 of adulthood. n = 101 worms for ctrl RNAi and 92 worms for *alg-1* RNAi. **(b)** Survival on paraquat (8 mM) of WT worms exposed to *alg-1* or control RNAi (empty vector) since day 0 of adulthood. n = 133 worms for ctrl RNAi and 138 worms for *alg-1* RNAi. **(c-d)** Relative GFP fluorescence of worms expressing **(c)** *sod-3p::gfp* (*muIs84*) (n = 16, 24, 16, 20 worms per condition) or **(d)** *gst-4p::gfp* (*dvIs19 III*) (n = 28, 23, 24, 15 worms per condition) exposed to *alg-1* or control RNAi (empty vector) since day 0 of adulthood. Worms were transferred to vehicle or 4 mM paraquat (PQ) plates on day 0 and fluorescence was measured on day 3. **(a-b)** Data were compared using the log-rank test. **** $P < 0.0001$. Representative data from three independent replicates. **(c-d)** Bars represent mean ± SEM. Comparisons were made using two-way ANOVA with Sidak's post hoc test. Combined data of two independent replicates. ns – non-significant ($P > 0.05$), ** $P < 0.01$, *** $P < 0.001$, **** $P < 0.0001$. Source data and exact P values (whenever available) are provided as a Source Data file.

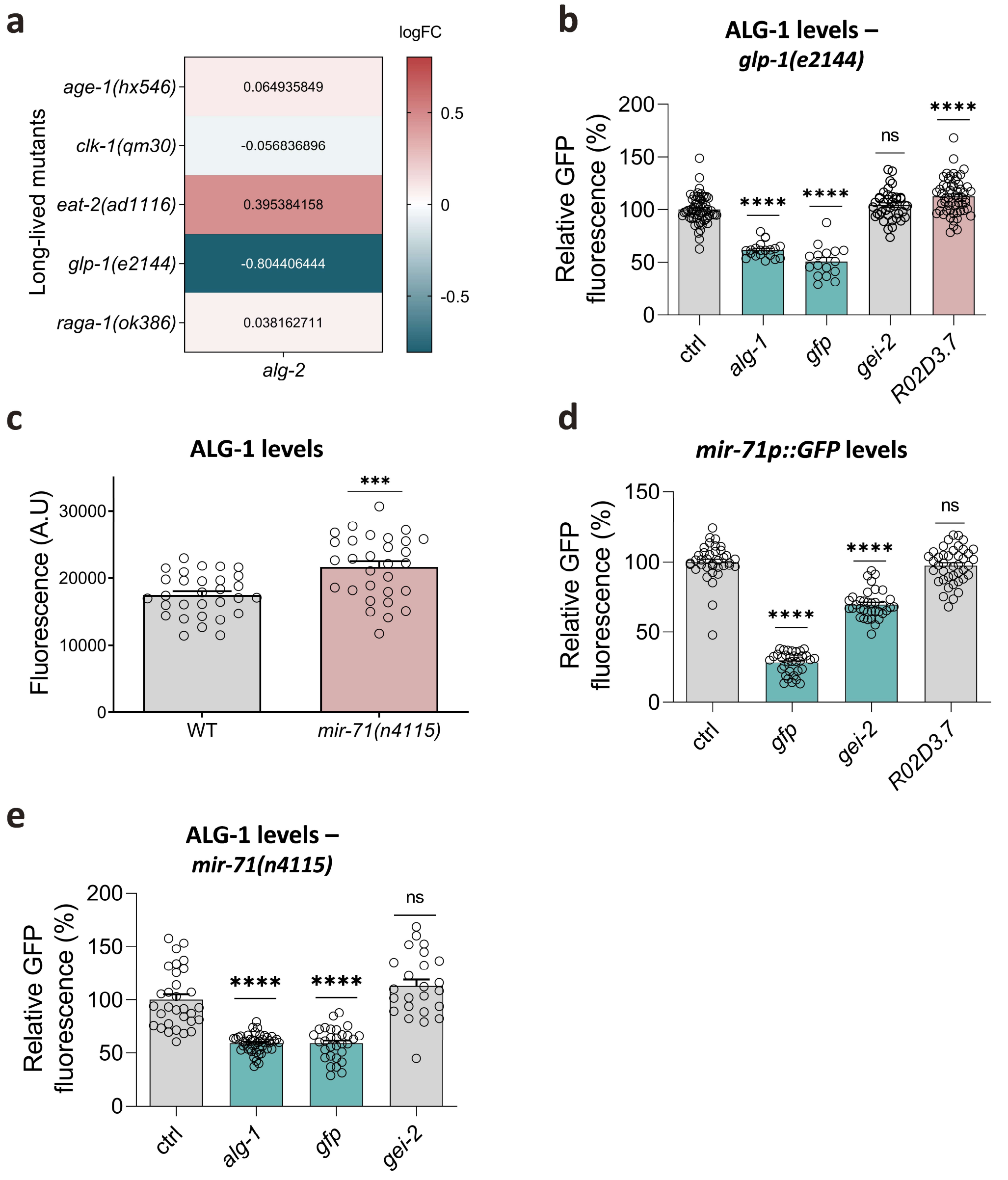

Supplementary Figure 2. ALG-1 expression is controlled in *cis* and *trans*. (a) *alg-2* gene expression in long-lived mutants as determined by RNAseq. Adjusted p-values and detailed information are described in Supplementary Dataset 1. **(b)** Relative GFP fluorescence of the *alg-1p::gfp::alg-1(zaIs5)* reporter in *glp-1(e2144)* mutants after RNAi exposure. *n* = 60, 20, 16, 39, 57 worms per condition. **(c)** Relative GFP fluorescence of the *alg-1p::gfp::alg-1(zaIs5)* reporter in *mir-71(n4115)* mutants. A.U represents the arbitrary unit of the mean fluorescence. *n* = 30 worms per condition. Statistics were determined by two-tailed paired Student's t-test. *** *P* < 0.001. **(d)** Relative GFP fluorescence of the *mir-71p::GFP (maIs352)* reporter strain after RNAi exposure. *n* = 38, 36, 37, 42 worms per condition. **(e)** Relative GFP fluorescence of the *alg-1p::gfp::alg-1(zaIs5)* reporter in *mir-71(n4115)* mutants after RNAi exposure. *n* = 32, 47, 30, 24 worms per condition. **(b-e)** Worms were grown on plates with control (ctrl) RNAi (empty vector), transferred to respective RNAi plates at day 0, and had their fluorescence measured on day 3. Bars represent mean ± SEM. Combined data of three independent replicates. For **(b**, **d-e)**, comparisons were made using one-way ANOVA with Dunnett's post hoc test. ns – non-significant (*P* > 0.05), **** *P* < 0.0001. Source data and exact *P* values (whenever available) are provided as a Source Data file.

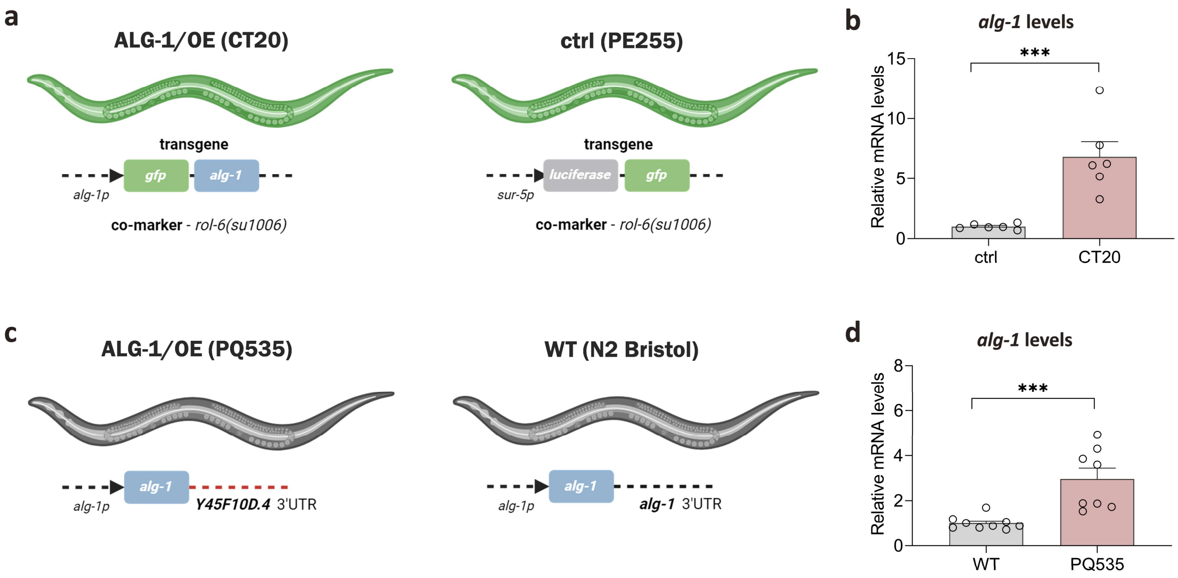

Supplementary Figure 3. **ALG-1 overexpression models.** **(a)** CT20: a strain generated by genomic insertion of the transgene *zaIs5[alg-1p::GFP::alg-1 + pRF4(rol-6(su1006))]*, which produces a functional GFP::ALG-1 fusion protein[25]. As a control (ctrl), we used PE255, a strain that carries the transgene *feIs5[sur-5p::luciferase::GFP + rol-6(su1006)]* integrated in the genome. This transgene overexpresses a *luciferase::GFP* fusion protein. Created with BioRender.com. **(b)** Relative mRNA expression of *alg-1* in ctrl (PE255) *vs.* CT20, measured by RT-qPCR on day 1 of adulthood. *n* = 6 pool of worms per condition. **(c)** PQ535: a strain in which the 3'UTR of *alg-1* is substituted by the 3'UTR of *Y45F10D.4* using CRISPR/Cas9 editing, thus creating the *ap428* allele (*alg-1::Y45F10D.4 3'UTR*) which overexpresses ALG-1[17]. In this case, the wild type (WT) N2 Bristol was used as a control. Created with BioRender.com. **(d)** Relative mRNA expression of *alg-1* in WT *vs.* PQ535, measured by RT-qPCR on day 1 of adulthood. *n* = 9 and 8 pools of worms per condition. **(b** and **d)** Data are the mean ± SEM from two independent replicates. Statistics were determined by two-tailed paired Student's t-test. ** $P < 0.01$, **** $P < 0.0001$. Source data and exact *P* values (whenever available) are provided as a Source Data file.

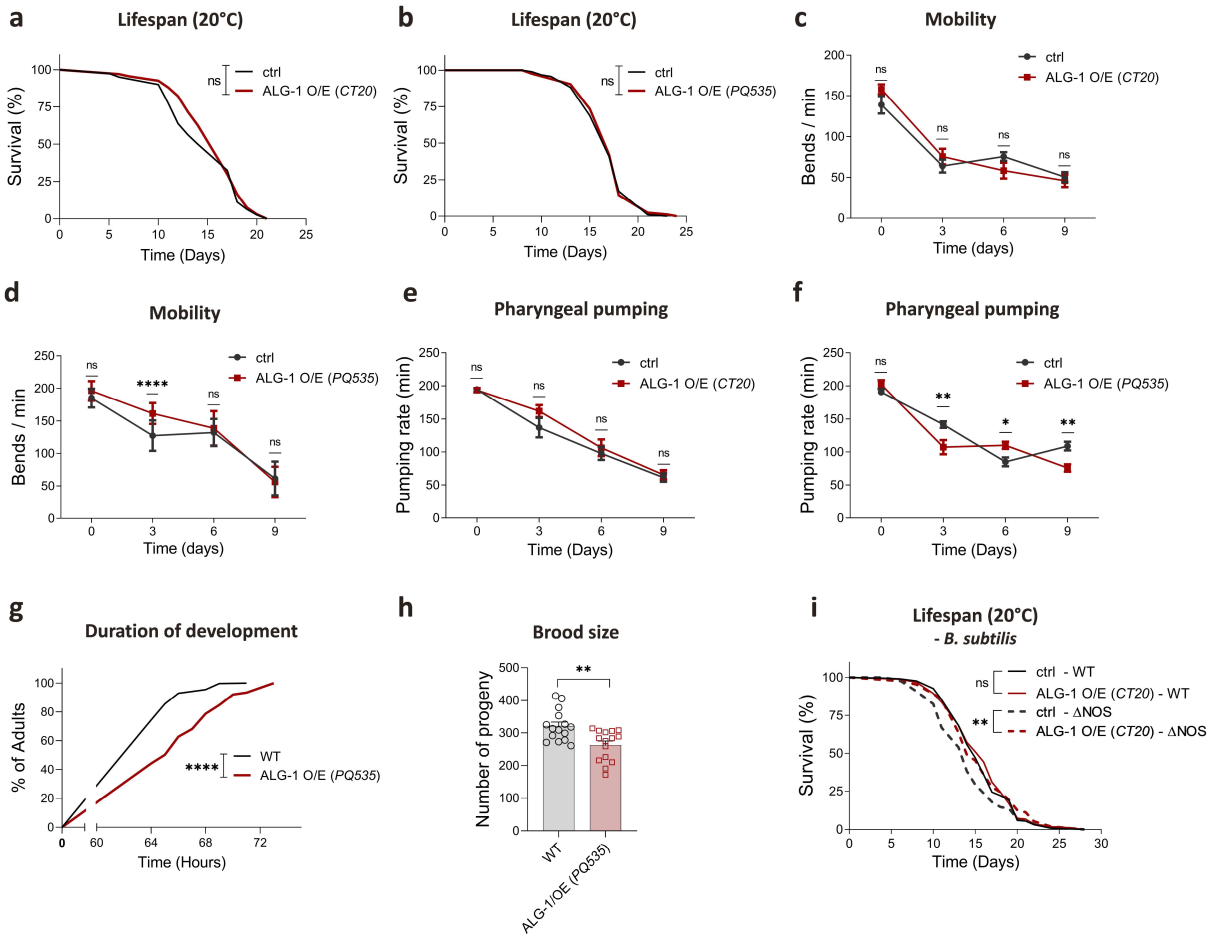

Supplementary Figure 4. ALG-1 overexpression does not affect lifespan or health span parameters, but delays development and reduces fertility in *C. elegans*. (a-b) Lifespan at 20°C of **(a)** CT20 (*n* = 80 worms for ctrl and 67 worms for CT20) and **(b)** PQ535 (*n* = 104 worms for WT and 95 worms for PQ535) on OP50-1 bacteria in comparison with their respective controls. **(c-d)** Mobility expressed as body bends per minute using **(c)** CT20 (*n* = 15 worms for each time point and strain) and **(d)** PQ535 (*n* = 15 worms for each time point and strain) strains in comparison with their controls. Representative data from two independent replicates. **(e-f)** Pharyngeal pumping per minute using **(e)** CT20 (*n* =15 worms for each time point and strain) and **(f)** PQ535 (*n* = 15 worms for each time point and strain) strains in comparison with their controls. Representative data from two independent replicates. **(g)** Duration of development of wild type (WT) *vs* ALG-1/OE (PQ535) worms on OP50-1 bacteria. Representative data from three independent biological replicates. *n* = 128 worms for WT and 194 worms for PQ535**. (h)** Brood size. *n* = 15 worms per condition. Bars represent mean ± SEM. Statistics were determined by two-tailed paired Student's t-test. ** *P* < 0.01 **(i)** Lifespan at 20°C on ΔNOS *B. subtilis* bacteria (nitric oxide-free). *n* = 114 worms for ctrl WT, 114 worms for ALG-1/OE WT, 111 worms for ctrl ΔNOS, 86 worms for ALG-1/OE ΔNOS. Representative data from two independent replicates. **(a-b, g** and **i)** Data were compared using the log-rank test. ns – non-significant (*P* > 0.05), * *P* < 0.05, ** *P* < 0.01, **** *P* < 0.0001. **(c-f)** Bars represent mean ± SEM. Comparisons were made using two-way ANOVA with Sidak's post hoc test. ns – non-significant (*P* > 0.05), * *P* < 0.05, ** *P* < 0.01, **** *P* < 0.0001. Source data and exact *P* values (whenever available) are provided as a Source Data file.

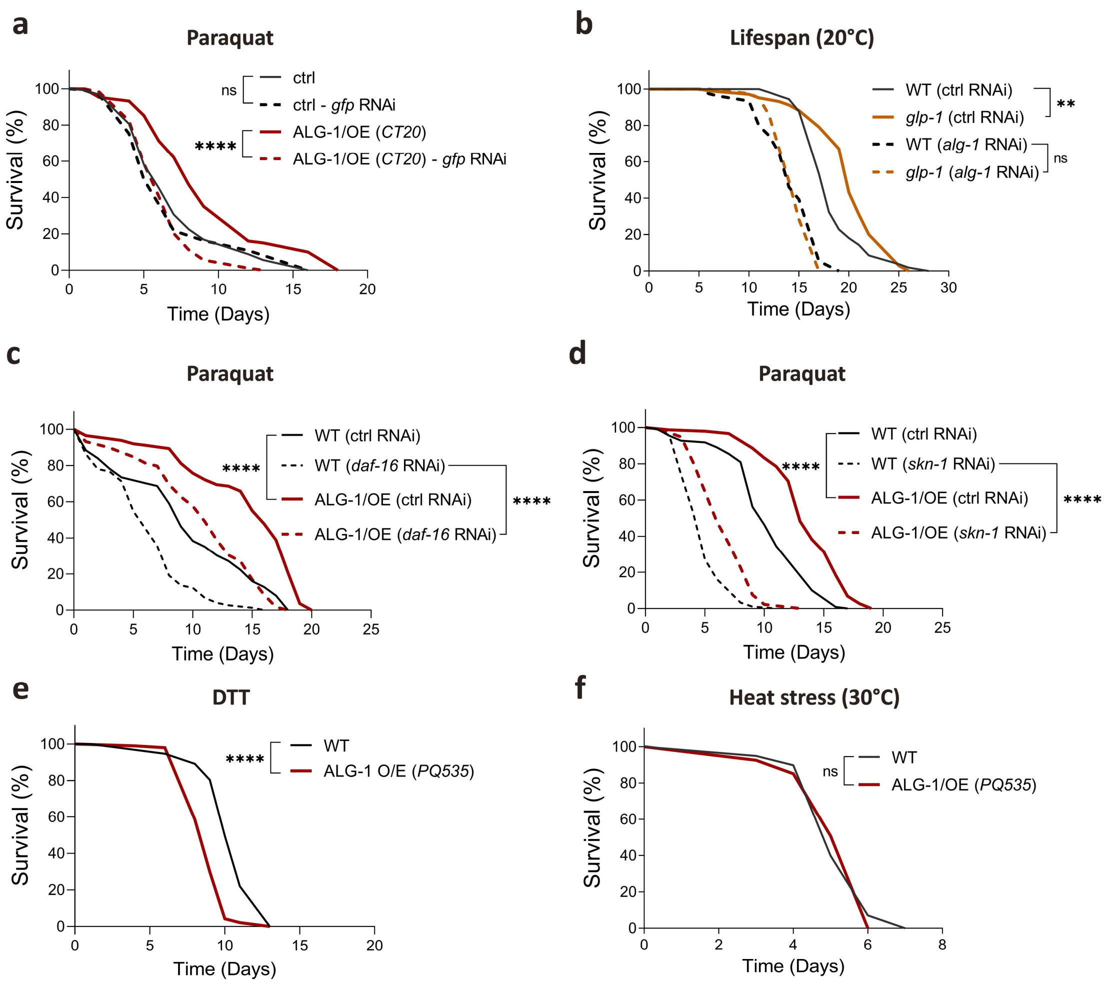

Supplementary Figure 5. ALG-1 overexpression promotes resistance to oxidative stress, but not to other types of stressors. (a) Survival on paraquat (8 mM). ALG-1/OE (CT20) and control (ctrl; PE255) worms were treated with *gfp* or control (ctrl) RNAi (empty vector) from day 0. *n* = 133 worms for ctrl, 123 worms for ctrl *gfp* RNAi, 142 worms for CT20, 125 worms for CT20 *gfp* RNAi. Representative data from three independent replicates. **(b)** Lifespan of *glp-1(e2144) vs* WT worms exposed to *alg-1* or control RNAi (empty vector) since day 0 of adulthood. *n* = 130 worms for WT ctrl RNAi, 113 worms for WT *alg-1* RNAi, 98 worms for *glp-1(e2144)* ctrl RNAi, 145 worms for *glp-1(e2144) alg-1* RNAi. Representative data from three independent replicates. **(c)** Survival on paraquat (8 mM). ALG-1/OE (CT20) and ctrl worms were exposed to *daf-16* or control (ctrl) RNAi (empty vector) from day 0 of adulthood. *n* = 69 worms for WT ctrl RNAi, 73 worms for WT *daf-16* RNAi, 114 worms for ALG-1/OE ctrl RNAi, 60 worms for ALG-1/OE *daf-16* RNAi. Representative data from two independent replicates. **(d)** Survival on paraquat (8 mM). ALG-1/OE (CT20) and ctrl (PE255) worms were exposed to *skn-1* or control (ctrl) RNAi (empty vector) from day 0 of adulthood. *n* = 91 worms for WT ctrl RNAi, 120 worms for WT *skn-1* RNAi, 113 worms for ALG-1/OE ctrl RNAi, 77 worms for ALG-1/OE *skn-1* RNAi. Representative data from three independent replicates. **(e)** Survival on DTT (5 mM) of ALG-1/OE (PQ535) worms in comparison with WT. Representative data from two independent replicates. *n* = 94 worms for WT and 96 worms for PQ535. **(f)** Survival on OP50-1 at 30°C of ALG-1/OE (PQ535) worms in comparison with WT. Representative data from two independent replicates. *n* = 147 worms for WT and 97 worms for PQ535. For all data, statistics were made using the log-rank test. ns – non-significant ($P > 0.05$), ** $P < 0.01$, **** $P < 0.0001$. Source data and exact *P* values (whenever available) are provided as a Source Data file.

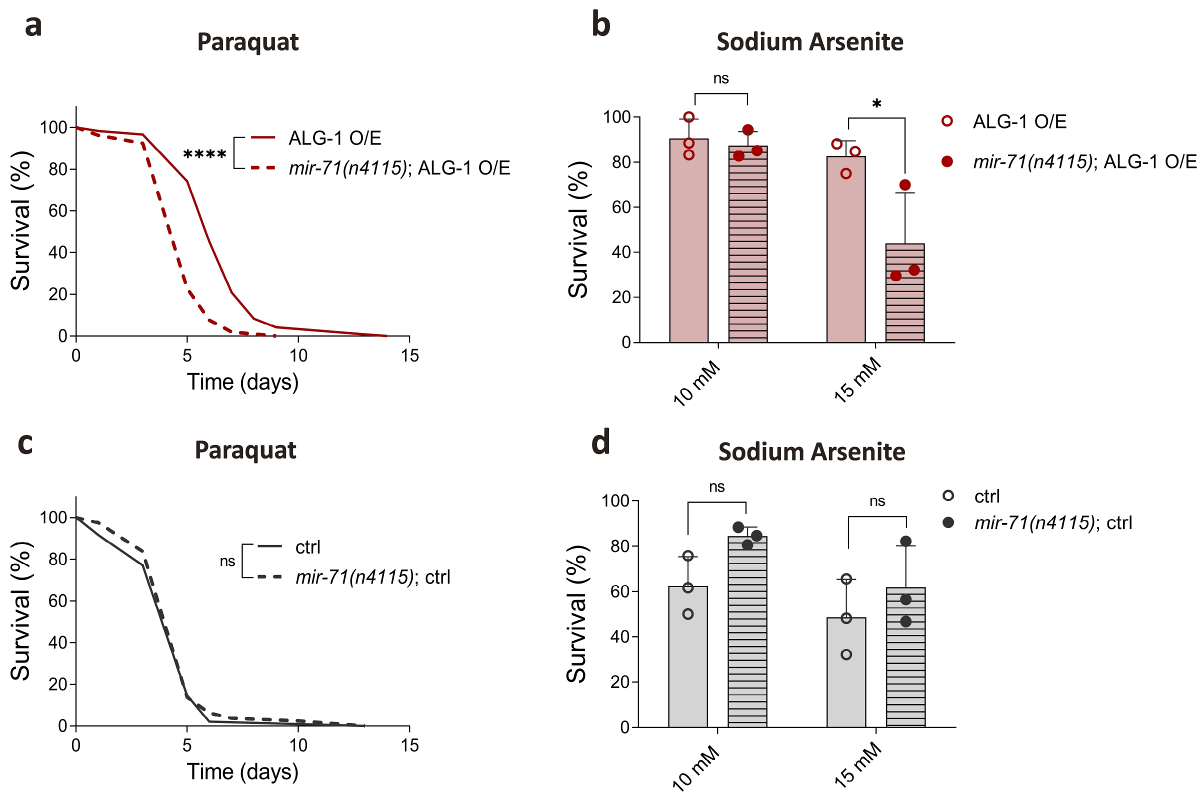

Supplementary Figure 6. Loss of miR-71 suppresses oxidative stress resistance of ALG-1/OE worms. (a) Survival on paraquat (4 mM) of ALG-1/OE (CT20) worms carrying or not the *mir-71(n4115)* mutation. *n* = 120 worms for ALG-1/OE and 106 worms for *mir-71;* ALG-1/OE. **(b)** Survival after 8 hours of exposure to sodium arsenite on day 3 of adulthood of ALG-1/OE (CT20) worms carrying or not the *mir-71(n4115)* mutation. *n* = 3 wells with 10 worms each per condition. **(c)** Survival on paraquat (4 mM) of control (ctrl) worms (PE255) carrying or not the *mir-71(n4115)* mutation. *n* = 84 worms for ctrl and 106 worms for *mir-71;* ctrl. **(d)** Survival after 8 hours of exposure to sodium arsenite on day 3 of adulthood of ctrl worms (PE255) carrying or not the *mir-71(n4115)* mutation. *n* = 3 wells with 10 worms each per condition. **(a, c)** Worms were grown on OP50-1 bacteria and transferred to vehicle or paraquat plates at the L4 stage. Representative data from three independent replicates. Data were compared using the log-rank test. ns – non-significant ($P > 0.05$), **** $P < 0.0001$. **(b, d)** Bar represent mean ± SEM. Comparisons were made using two-way ANOVA with Sidak's post hoc test. Representative data from three independent replicates. ns – non-significant ($P > 0.05$), * $P < 0.05$. Source data and exact *P* values (whenever available) are provided as a Source Data file.

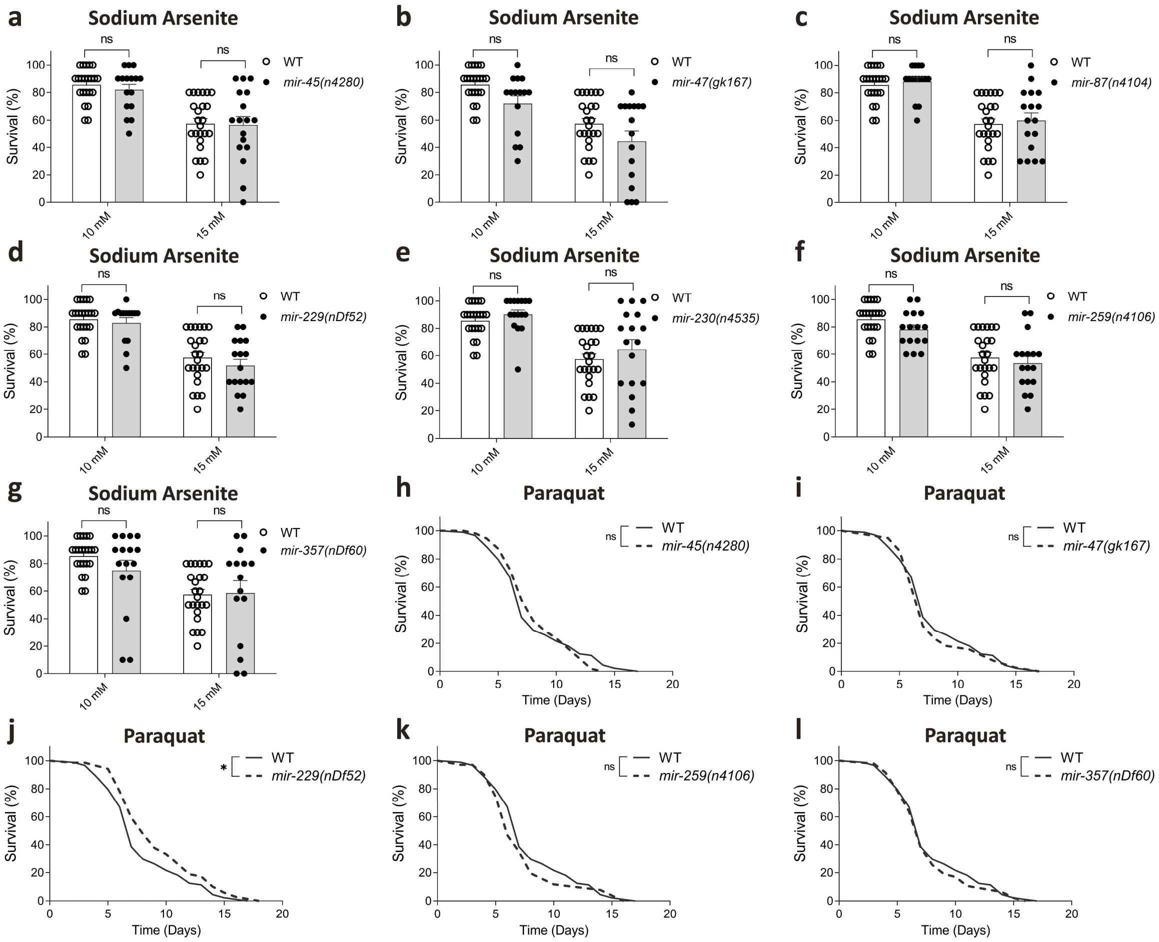

Supplementary Figure 7. Survival of miRNA null mutants on pro-oxidants. (a-g) Survival after 8 hours of exposure to sodium arsenite on day 3 of adulthood of **(a)** *mir-45(n4280)* (*n* = 22, 16, 24, 17 wells with 10 worms each per condition), **(b)** *mir-47(gk167)* (*n* = 22, 15, 24, 16 wells with 10 worms each per condition), **(c)** *mir-87(n4104)* (*n* = 22, 15, 24, 17 wells with 10 worms each per condition), **(d)** *mir-229(nDf52)* (*n* = 22, 14, 24, 17 wells with 10 worms each per condition), **(e)** *mir-230(n4535)* (*n* = 22, 16, 24, 17 wells with 10 worms each per condition), **(f)** *mir-259(n4106)* (*n* = 22, 16, 24, 17 wells with 10 worms each per condition), and **(g)** *mir-357(nDf60)* (*n* = 22, 16, 24, 15 wells with 10 worms each per condition) mutants *vs.* WT. Bars represent mean ± SEM. Combined data from three independent replicates. Comparisons were made using two-way ANOVA with Sidak's post hoc test. ns – non-significant ($P > 0.05$). **(h-l)** Survival on paraquat (4mM) of **(h)** *mir-45(n4280)*, **(i)** *mir-47(gk167)*, **(j)** *mir-229(nDf52)*, **(k)** *mir-259(n4106)*, and **(l)** *mir-357(nDf60)* mutants *vs.* WT worms. *n* = 119 worms for WT, 130 worms for *mir-45,* 104 worms for *mir-47*, 133 worms for *mir-229,* 149 worms for *mir-259,* 91 worms for *mir-357.* Data represent one experiment from three independent replicates. Experiments were performed together and hence share the same controls. They were split in different panels to allow better visualization. Comparisons were made using log-rank test. ns – non-significant ($P > 0.05$), * $P < 0.05$. Source data and exact *P* values (whenever available) are provided as a Source Data file.

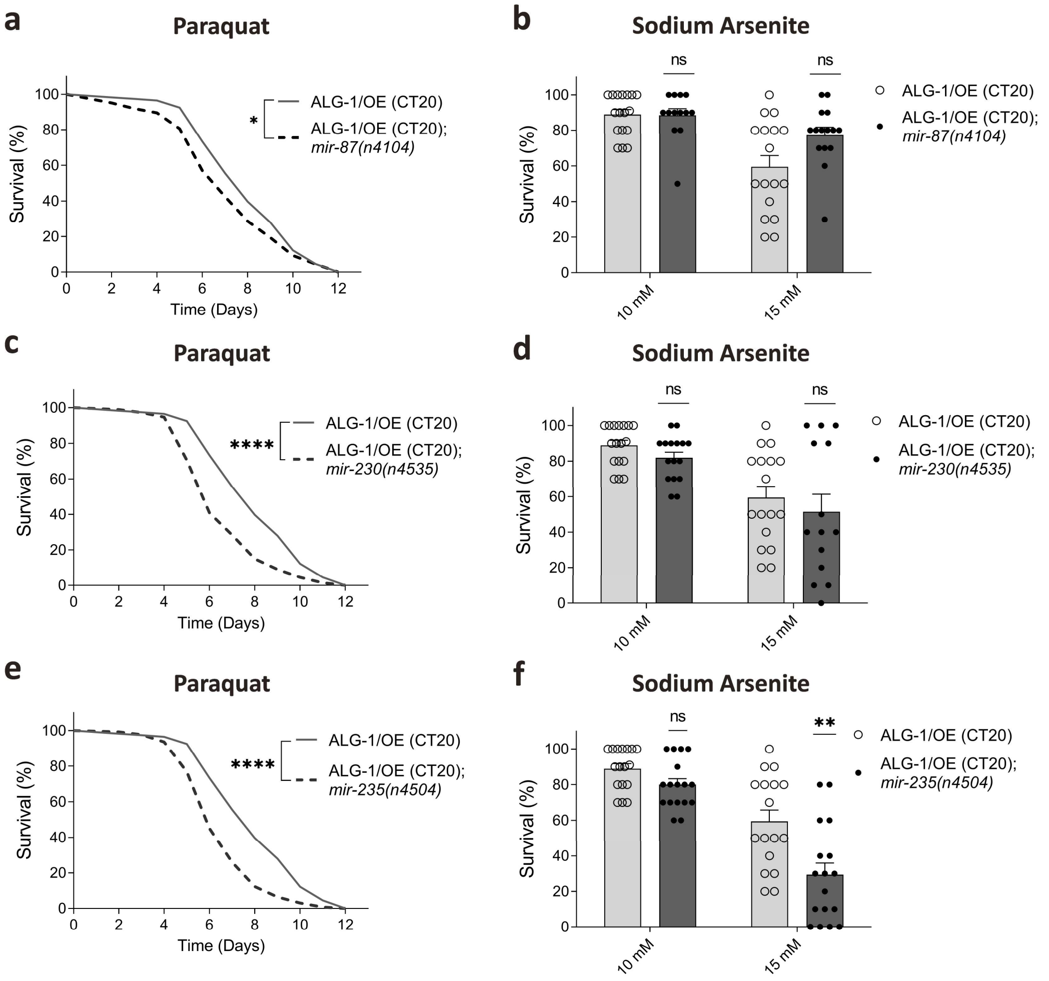

Supplementary Figure 8. *mir-87*, *mir-230*, and *mir-235* null mutations reduce oxidative stress resistance of ALG-1/OE worms. (a-b) ALG-1/OE worms carrying or not the *mir-87(n4104)* mutation. Survival on **(a)** paraquat (4mM) (*n* = 116 worms for ALG-1/OE and 117 worms for ALG-1/OE; *mir*-87) and survival after 8 hours of exposure to **(b)** sodium arsenite (*n* = 17,14,17,15 wells with 10 worms each per condition) on day 3 of adulthood. **(c-d)** ALG-1/OE worms carrying or not the *mir-230(n4535)* mutation. Survival on **(c)** paraquat (4mM) (*n* = 116 worms for ALG-1/OE and 179 worms for ALG-1/OE; *mir*-230) and survival after 8 hours of exposure to **(d)** sodium arsenite (*n* = 17,16,17,14 wells with 10 worms each per condition) on day 3 of adulthood. **(e-f)** ALG-1/OE worms carrying or not the *mir-235(n4504)* mutation. Survival on **(e)** paraquat (4 mM) (*n* = 116 worms for ALG-1/OE and 140 worms for ALG-1/OE; *mir*-235) and survival after 8 hours of exposure to **(f)** sodium arsenite (*n* = 17 wells with 10 worms each per condition) on day 3 of adulthood. **(a,c** and **e).** Data were compared using the log-rank test and represent one experiment from three independent replicates. * $P < 0.05$, **** $P < 0.0001$. **(b, d** and **f)** Bars represent mean ± SEM. Comparisons were made using two-way ANOVA with Sidak's post hoc test. ns – non-significant ($P > 0.05$), ** $P < 0.01$. Combined data from three independent replicates. Experiments were performed together and hence share the same controls. Source data and exact *P* values (whenever available) are provided as a Source Data file.

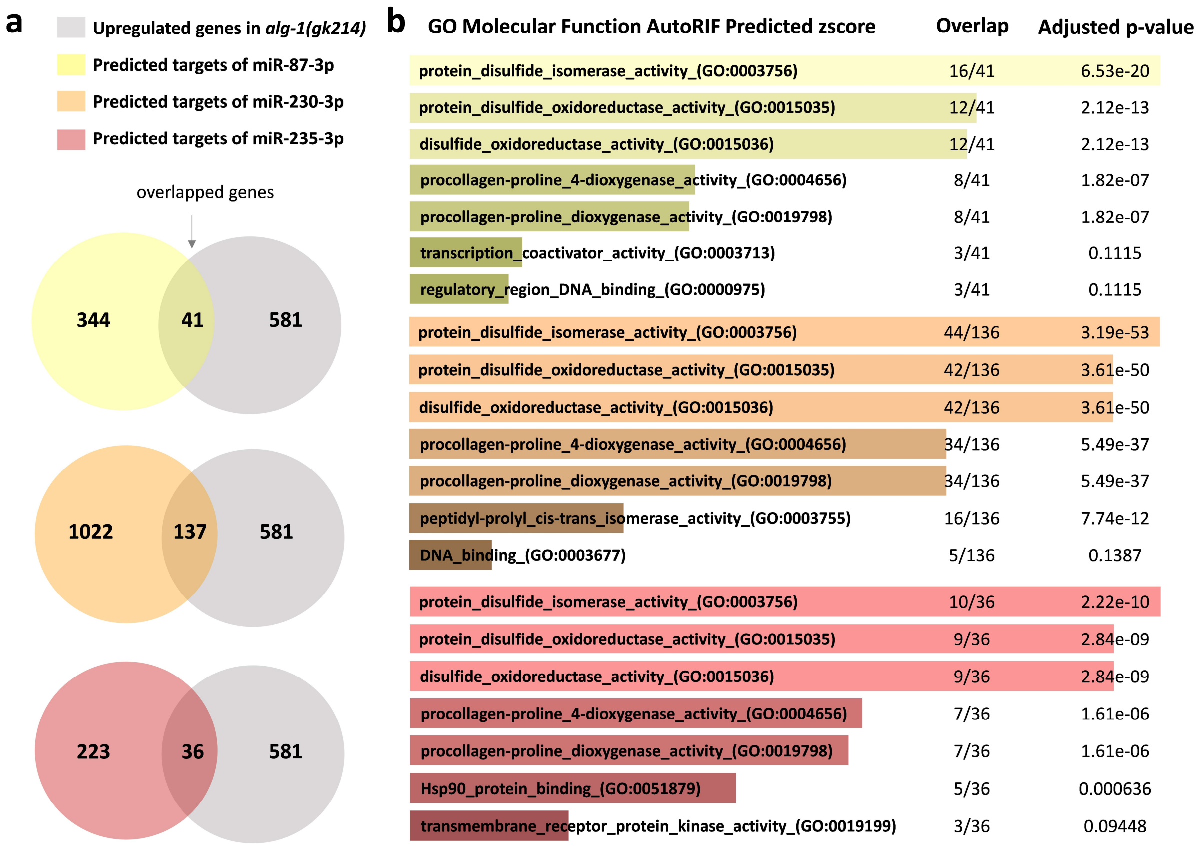

Supplementary Figure 9. Genes associated with the PDI pathway are enriched among the putative targets of miRNAs upregulated after ALG-1 overexpression. (a) Venn diagrams showing the overlapped genes of predicted targets of miRNAs (yellow - miR-87-3p, orange – miR-230-3p, and red – miR-235-3p) with genes upregulated in the *alg-1(gk214)* mutants (gray circles). **(b)** Gene ontology of the overlapped genes based on their enrichment among the gene sets found in the GO Molecular Function database of WormEnrichR according to their AutoRIF (gene-publication associations) predicted Z score. The target list of each miRNA is available in Supplementary Dataset 4.

Supplementary Table 1. ***C. elegans*** **strains used in the study.**

Strain name	Genotype	Description
**N2 Bristol**	-	Wild type
**CT20**	*zaIs5 [alg-1p::GFP::alg-1 + rol-6(su1006)]*	ALG-1 GFP reporter and ALG-1/OE
**PE255**	*feIs5 [sur-5p::luciferase::GFP + rol-6(su1006)] X*	ctrl for CT20
**CF1903**	*glp-1(e2144) III.*	*glp-1* mutant
**MT12993**	*mir-71(n4115) I*	*mir-71* knockout
**MT13433**	*mir-45(n4280) II*	*mir-45* knockout
**VC328**	*mir-47(gk167) X*	*mir-47* knockout
**MT12958**	*mir-87(n4104) V*	*mir-87* knockout
**MT14662**	*mir-230(n4535) X*	*mir-230* knockout
**MT17997**	*mir-235(n4504) I*	*mir-235* knockout
**MT12969**	*mir-259(n4106) V*	*mir-259* knockout
**MT15019**	*nDf60 V*	*mir-357* knockout
**MAM44**	*glp-1(e2144) III; zaIs5[alg-1p::GFP::alg-1 + rol-6(su1006)]*	*glp-1;* ALG-1 GFP reporter (CT20)
**MAM131**	*mir-71(n4115); zaIs5[alg-1p::GFP::alg-1 + rol-6(su1006)]*	*mir-71* KO; ALG-1 GFP reporter (CT20)
**VT2084**	*maIs352 [mir-71p::GFP + unc-119(+)]*	*mir-71* GFP reporter
**PQ535**	*alg-1(ap428 [alg-1::Y45F10D.4 3'UTR]) X.*	ALG-1/OE
**PQ530**	*alg-1(ap423[3xflag::gfp::alg-1]) X.*	ALG-1 GFP reporter
**WBM1610**	*alg-1(ap428 [alg-1::Y45F10D.4 3'UTR]) X +* N2; *dvIs19 III.*	ALG-1/OE (PQ535); *gst-4* GFP reporter
**WBM1612**	*muIs84 [(pAD76) sod-3p::GFP + rol-6(su1006)]; alg-1(ap428 [alg-1::Y45F10D.4 3'UTR]) X.*	ALG-1/OE (PQ535); *sod-3* GFP reporter
**WBM1619**	*alg-1(ap423[3xflag::gfp::alg-1]) X; glp-1(e2144) III.*	*glp-1* mutant*;* ALG-1 GFP reporter (PQ530)
**WBM1623**	*alg-1(ap423[3xflag::gfp::alg-1]) X; clk-1(qm30) III*	*clk-1* mutant*;* ALG-1 GFP reporter (PQ530)
**WBM1626**	*alg-1(ap423[3xflag::gfp::alg-1]) X; eat-2(ad1116) II*	*eat-2* mutant*;* ALG-1 GFP reporter (PQ530)
**WBM1625**	*alg-1(ap423[3xflag::gfp::alg-1]) X; raga-1(ok386) II.*	*raga-1* mutant*;* ALG-1 GFP reporter (PQ530)
**CF1553**	*muIs84. (sod-3p::gfp)*	*sod-3* GFP reporter
**WBM552**	N2; *dvIs19 III.*	*gst-4* GFP reporter
