# Supplementary material for: An Intricate Network Involving the Argonaute ALG-1 Modulates Organismal Resistance to Oxidative Stress
Source: Nat Commun. 2024 Apr 9;15:3070. doi: 10.1038/s41467-024-47306-4 (PMC11003958; doi:10.1038/s41467-024-47306-4)
Supplement: Supplementary file 3 — Description of Additional Supplementary Files [file 41467_2024_47306_MOESM3_ESM.pdf]

Description of Additional Supplementary Files:

Supplementary Dataset 1. *p-adj* values of the RNAseq analysis.

Supplementary Dataset 2. Gene names of transcription factors used in the RNAi screen and *alg-1* reporter levels upon RNAi silencing.

Supplementary Dataset 3. Differentially expressed miRNAs according with the small RNAseq data.

Supplementary Dataset 4. List of miRNA targets predicted by TargetScan and overlap with genes upregulated in *alg-1(gk214)* mutants.
