# Supplementary material for: An Intricate Network Involving the Argonaute ALG-1 Modulates Organismal Resistance to Oxidative Stress
Source: Nat Commun. 2024 Apr 9;15:3070. doi: 10.1038/s41467-024-47306-4 (PMC11003958; doi:10.1038/s41467-024-47306-4)
Supplement: Supplementary file 8 — Reporting Summary [file 41467_2024_47306_MOESM8_ESM.pdf]

nature portfolio

Corresponding author(s): Marcelo A. Mori

Last updated by author(s): Mar 9, 2024

Reporting Summary

Nature Portfolio wishes to improve the reproducibility of the work that we publish. This form provides structure for consistency and transparency in reporting. For further information on Nature Portfolio policies, see our Editorial Policies and the Editorial Policy Checklist.

Statistics

For all statistical analyses, confirm that the following items are present in the figure legend, table legend, main text, or Methods section.

n/a	Confirmed	
☐	☒	The exact sample size (*n*) for each experimental group/condition, given as a discrete number and unit of measurement
☐	☒	A statement on whether measurements were taken from distinct samples or whether the same sample was measured repeatedly
☐	☒	The statistical test(s) used AND whether they are one- or two-sided<br>*Only common tests should be described solely by name; describe more complex techniques in the Methods section.*
☒	☐	A description of all covariates tested
☐	☒	A description of any assumptions or corrections, such as tests of normality and adjustment for multiple comparisons
☐	☒	A full description of the statistical parameters including central tendency (e.g. means) or other basic estimates (e.g. regression coefficient) AND variation (e.g. standard deviation) or associated estimates of uncertainty (e.g. confidence intervals)
☐	☒	For null hypothesis testing, the test statistic (e.g. *F*, *t*, *r*) with confidence intervals, effect sizes, degrees of freedom and *P* value noted<br>*Give P values as exact values whenever suitable.*
☒	☐	For Bayesian analysis, information on the choice of priors and Markov chain Monte Carlo settings
☒	☐	For hierarchical and complex designs, identification of the appropriate level for tests and full reporting of outcomes
☒	☐	Estimates of effect sizes (e.g. Cohen's *d*, Pearson's *r*), indicating how they were calculated

Our web collection on statistics for biologists contains articles on many of the points above.

Software and code

Policy information about availability of computer code

Data collection	The modENCODE ChIP-Sequencing consortium (v33) was utilized to collect transcription factor candidates. TargetScanWorm (Release 6.2) was employed to gather candidate targets of miRNAs. WormExp (v2.0) was used to conduct the intersection of candidate targets of miRNAs with genes upregulated in RNA-seq analysis of alg-1 loss of function mutants.
Data analysis	GraphPad Prism (v8.4.3) was employed for statistical analysis, while R (v1.3.1) was utilized to conduct the differential expression analysis of RNA-seq data. Fluorescence quantification was performed using ImageJ (v1.53q), and gene ontology analysis was carried out using WormEnrichr (ModEnrichr toolkit - August 1, 2018).

For manuscripts utilizing custom algorithms or software that are central to the research but not yet described in published literature, software must be made available to editors and reviewers. We strongly encourage code deposition in a community repository (e.g. GitHub). See the Nature Portfolio guidelines for submitting code & software for further information.

Data

Policy information about availability of data

All manuscripts must include a data availability statement. This statement should provide the following information, where applicable:

- Accession codes, unique identifiers, or web links for publicly available datasets
- A description of any restrictions on data availability
- For clinical datasets or third party data, please ensure that the statement adheres to our policy

All data generated or analyzed during this study are provided in the Supplementary Information and Source Data files and are available in public repositories or upon request from their original sources. The small RNA sequencing data generated in this study (Fig. 4a-e) have been deposited in the GEO database under accession code GSE260938 [https://www.ncbi.nlm.nih.gov/geo/query/acc.cgi?acc=GSE260938]. The processed data is also available in the Supplementary Dataset 3. The RNA sequencing data re-used in this study (Fig. 1a,g and Supplementary Fig. 2a) are available in the GEO database under accession code GSE111338 [https://www.ncbi.nlm.nih.gov/geo/query/acc.cgi?acc=GSE111338] or were shared by and is available upon request from the Mair lab. The processed data used in the study are also available in the Supplementary Dataset 1. The microarray data re-used in this study (Fig. 5a and Supplementary Fig. 9) are available in the GEO database under accession code GSE19138 [https://www.ncbi.nlm.nih.gov/geo/query/acc.cgi?acc=GSE19138]. The processed data used in this study is also available in the Supplementary Dataset 4.

Research involving human participants, their data, or biological material

Policy information about studies with human participants or human data. See also policy information about sex, gender (identity/presentation), and sexual orientation and race, ethnicity and racism.

Reporting on sex and gender	No human participants were involved in this study.
Reporting on race, ethnicity, or other socially relevant groupings	No human participants were involved in this study.
Population characteristics	No human participants were involved in this study.
Recruitment	No human participants were involved in this study.
Ethics oversight	No human participants were involved in this study.

Note that full information on the approval of the study protocol must also be provided in the manuscript.

Field-specific reporting

Please select the one below that is the best fit for your research. If you are not sure, read the appropriate sections before making your selection.

☒ Life sciences ☐ Behavioural & social sciences ☐ Ecological, evolutionary & environmental sciences

For a reference copy of the document with all sections, see nature.com/documents/nr-reporting-summary-flat.pdf

Life sciences study design

All studies must disclose on these points even when the disclosure is negative.

Sample size	The choice for sample size for each assay was based on previous relevant publications by our group. For survival analysis, approximately 120 worms per condition were used to achieve a final sample size of around 100, considering potential loss of animals during the experiment (Pinto et al., Redox Biology, 2018 - doi: 10.1016/j.redox.2018.06.006). For sodium arsenite stress resistance assays, 50-100 worms per group were used (Mori et al., Cell Metabolism, 2012 - doi: 10.1016/j.cmet.2012.07.017). For fluorescence measurements, at least 10 worms per group were considered based on a previous publication (De-Souza et al., Nucleic Acid Res 2019 - doi: 10.1093/nar/gkz154). For gene expression analysis, RNA from pools of 100 adult worms was used, while for small RNA-seq analysis, pools of 500-800 adult worms were used (Pinto et al., Redox Biology, 2018 - doi: 10.1016/j.redox.2018.06.006). Sample sizes for pharyngeal pumping, mobility, and brood size assays were also guided by a previous publication (Pinto et al., Redox Biology, 2018 - doi: 10.1016/j.redox.2018.06.006).
Data exclusions	No data was excluded.
Replication	At least two independent experiments were carried out with multiple worms in each experiment. The number of experiments conducted and the number of worms used in each experiment are detailed in the figure legends.
Randomization	To achieve randomization, hundreds (300 to 1000 worms) of age-synchronized C. elegans were randomly allocated to designated experimental plates (for survival, brood size, fluorescence measurements, and gene expression analysis) or wells (for sodium arsenite resistance, pharyngeal pumping, and mobility assays).
Blinding	Many assays in this study relied on fully- or semi-automated data acquisition (e.g., qPCR machine, sequencing system, image analysis software, gene ontology/miRNA targets curation engines), thereby minimizing researcher bias. For non-automated assays, we conducted

different biological replicates with different researchers analyzing the data whenever possible. Moreover, these non-automated experiments, such as survival assays, pharyngeal pumping, mobility, and brood size measurements were based on binary outcomes (living/dead, bend/static, pump/static, eggs/no eggs on the plate surface), which minimizes the possibility of unconscious bias. Considering these factors, blinding was not implemented.

Reporting for specific materials, systems and methods

We require information from authors about some types of materials, experimental systems and methods used in many studies. Here, indicate whether each material, system or method listed is relevant to your study. If you are not sure if a list item applies to your research, read the appropriate section before selecting a response.

Materials & experimental systems

n/a	Involved in the study
☒	☐ Antibodies
☐	☒ Eukaryotic cell lines
☒	☐ Palaeontology and archaeology
☐	☒ Animals and other organisms
☒	☐ Clinical data
☒	☐ Dual use research of concern
☒	☐ Plants

Methods

n/a	Involved in the study
☒	☐ ChIP-seq
☒	☐ Flow cytometry
☒	☐ MRI-based neuroimaging

Eukaryotic cell lines

Policy information about cell lines and Sex and Gender in Research

Cell line source(s)	HEK293T (originally from ATCC)
Authentication	Cells were not authenticated, but HEK293T cells behaved as expected morphologically.
Mycoplasma contamination	Cells were not tested for mycoplasma contamination.
Commonly misidentified lines (See ICLAC register)	No commonly misidentified lines were used in this study.

Animals and other research organisms

Policy information about studies involving animals; ARRIVE guidelines recommended for reporting animal research, and Sex and Gender in Research

Laboratory animals	Organism: Caenorhabditis elegans.<br>All strains used in this study are listed in Supplementary Table 1.<br>We used worms of different stages and monitored them throughout lifespan.
Wild animals	No wild animal was used in this study.
Reporting on sex	We used hermaphrodites in all experiments and males only for mating.
Field-collected samples	No field-collected samples were used in this study.
Ethics oversight	This study did not involve materials or animal models that require ethical approval.

Note that full information on the approval of the study protocol must also be provided in the manuscript.

Plants

Seed stocks	No plants were used in this study.
Novel plant genotypes	No plants were used in this study.
Authentication	No plants were used in this study.
